# Association of MAGEA4 expression with molecular and immune subtypes and clinical outcomes in recurrent bladder cancer

**DOI:** 10.3389/fonc.2026.1906141

**Published:** 2026-10-15

**Authors:** HuaRong Luo, ChengDang Xu, YiFan Liu, YiWen Yao, ZuJun Sun, Yan Gu, TianRu Wang

**Affiliations:** 1 Department of Urology, Tongji Hospital of Tongji University, Shanghai, China; 2 School of Medicine, Tongji University, Shanghai, China; 3 Department of Laboratory Medicine, Tongji Hospital of Tongji University, Shanghai, China; 4 Department of Gastrointestinal Surgery, Tongji Hospital of Tongji University, Shanghai, China

**Keywords:** bladder cancer, immune subtyping, MAGEA4, molecular subtyping, prognosis, recurrence, RNA sequencing

## Abstract

**Background:**

This study assessed MAGEA4 expression in recurrent bladder cancer, examined its relationships with molecular and immune subtypes, and evaluated its association with treatment response and prognosis.

**Methods:**

This single-center exploratory cohort study enrolled 20 patients. RNA sequencing was performed on recurrent tumor tissue samples. The EU molecular subtypes were determined. Immune subtypes were classified, and PD-L1 and HER2 status were assessed. GO, KEGG, and Reactome enrichment analyses were performed. Patients were divided into MAGEA4-high and MAGEA4-low groups using the cohort median expression level. Differences in the composition of molecular/immune subtypes were compared. Within-cohort ROC analysis was used to assess discrimination of clinical benefit, and PFS was analyzed using Kaplan-Meier and Cox methods.

**Results:**

The top 20 genes with the most prominent between-subtype expression differences were identified. Genes upregulated in the Luminal papillary subtype were enriched in terms related to cell-cell junctions and epithelial structure maintenance. Downregulated genes were enriched in pathways associated with immune activation and inflammatory infiltration. Enrichment signals involved the PI3K-Akt, MAPK, neuroactive ligand-receptor interaction, and JAK-STAT pathways. Significant differences in PD-L1 status, TMIT subtypes, and EU subtypes were observed between MAGEA4-high and MAGEA4-low groups, and the six MAGEA4-immune/molecular association tests remained below q<0.05 after Benjamini-Hochberg correction. MAGEA4 expression also differed according to PD-L1 status, TMIT subtype, and binary EU subtype grouping. Within-cohort ROC analysis yielded AUC values of 0.798, 0.800, and 0.750 in the overall, immunotherapy, and combination-therapy cohorts, respectively; the bootstrap optimism-corrected overall AUC was 0.793. High MAGEA4 expression was associated with shorter PFS.

**Conclusion:**

MAGEA4 expression was associated with PD-L1 status, TMIT immune subtypes, and EU molecular subtypes, showed within-cohort discrimination of clinical benefit, and was associated with poorer PFS. These findings support further evaluation of MAGEA4 as a candidate molecular stratification marker in independent cohorts.

## Introduction

Bladder cancer is the most common malignancy of the urinary tract, ranking ninth in global cancer incidence rates. According to GLOBOCAN 2022 data, there were approximately 614,000 new cases and 221,000 deaths from bladder cancer worldwide in 2022, with the highest absolute number of cases observed in East Asia (1). In China, based on the latest report jointly released by the National Cancer Center and the International Agency for Research on Cancer (IARC), the age-standardized incidence rate of bladder cancer in 2022 was 3.44 per 100,000, and the mortality rate was 1.34 per 100,000 (2). Trend analysis based on Global Burden of Disease (GBD) 2019 data indicates that the age-standardized incidence rate of bladder cancer in China has been continuously increasing from 1990 (3.30 per 100,000) to 2019 (5.16 per 100,000) (average annual percent change [AAPC], 1.47). It is projected that the number of new cases will rise to approximately 150,000 by 2030, reflecting a persistently substantial disease burden (3).

Regarding pathological stage, approximately 75% of primary bladder cancers are non-muscle-invasive bladder cancer (NMIBC), encompassing stages Ta, T1, and Tis. Approximately 20%-25% are muscle-invasive bladder cancer (MIBC), with a 5-year survival rate of about 50%-60% following radical cystectomy combined with pelvic lymph node dissection. Approximately 5%-10% of patients present with metastatic disease at diagnosis, for whom platinum-based chemotherapy regimens confer a poor prognosis (4). However, even after standard initial treatment, bladder cancer exhibits a high propensity for recurrence. The overall recurrence rate for NMIBC within 5 years of treatment is 50%-80%, and approximately 30% of high-risk patients may progress to MIBC. This characteristic necessitates lifelong regular follow-up for bladder cancer patients (5). Currently, cystoscopy remains the gold standard for bladder cancer recurrence surveillance, offering direct visualization of intravesical lesions. However, its invasive nature leads to poor tolerance, and approximately 10% of cases are complicated by urinary tract infection. Furthermore, about 10%-20% of lesions may be missed by white-light cystoscopy, and adherence to intensive follow-up is difficult to maintain (5). Urine cytology, as an adjunctive non-invasive method, has a pooled sensitivity of only approximately 37% and specificity of approximately 95% according to meta-analyses. Its sensitivity is particularly low for low-grade lesions (approximately 16%), while it is relatively higher for high-grade tumors (approximately 84%). Moreover, results are significantly influenced by reader experience (6, 7). Therefore, additional biomarkers that improve biological characterization and clinical risk assessment remain relevant to the management of bladder cancer.

The emergence of molecular subtyping has provided new insights for precision diagnosis and treatment of bladder cancer. With advances in transcriptomic research, molecular classification systems for bladder cancer have been progressively established. Different molecular subtypes exhibit distinct gene expression profiles, tumor microenvironments, and clinical outcomes (8). Subsequently, Kamoun et al. proposed a consensus classification framework comprising six subtypes based on the integration of multiple independent classification systems: luminal papillary, luminal non-specified, luminal unstable, stroma-rich, basal/squamous, and neuroendocrine-like. This framework provides a unified reference for understanding bladder cancer heterogeneity and for research on subtype-guided precision therapy (9). Further studies have indicated that molecular subtyping not only helps elucidate the biological heterogeneity of bladder cancer but also holds potential clinical stratification value (10). For NMIBC, recent molecular and genomic studies have shown associations between different subtypes and risks of disease progression, treatment outcomes, and clinical behavior, potentially offering supplementary evidence for identifying high-risk patients and guiding individualized management. However, the clinical application of subtyping still requires further prospective validation (11–13).

Melanoma-associated antigen A4 (MAGEA4) belongs to the cancer-testis antigen (CTA) family. Its expression in normal tissues is strictly restricted to testicular germ cells and placental trophoblast cells, whereas it is widely aberrantly activated in various malignant tumors (14). In bladder cancer, MAGE-A4 protein expression increases significantly with rising tumor grade and stage and is independently associated with aggressive tumor phenotypes and poorer cancer-specific survival (15, 16). Furthermore, the CTA family exhibits a high overall expression rate in urothelial tumors, representing an important source for immunotherapeutic target development (17). However, the relationship of MAGEA4 expression with molecular subtypes, immune phenotypes, treatment response, and prognosis in recurrent bladder cancer remains incompletely characterized.

In summary, this study aims to analyze MAGEA4 expression in recurrent bladder cancer tissues, investigate its association with molecular and immune subtypes, and evaluate its relationship with treatment response and prognosis. The findings may provide molecular evidence for subtype-oriented characterization and treatment-response assessment while supporting further evaluation of MAGE-A4 in translational studies of bladder cancer.

## Materials and methods

### Study design and participants

This single-center exploratory cohort study enrolled patients with recurrent bladder cancer diagnosed and treated at the Tongji Hospital of Tongji University. Inclusion criteria were: (1) pathologically confirmed bladder cancer that had recurred, with recurrence determined by radiological assessment; (2) availability of recurrent tumor tissue specimens meeting requirements for transcriptome sequencing; (3) availability of relevant clinical data and follow-up information. Exclusion criteria were failure of sequencing due to poor tissue sample quality, or inability to evaluate outcomes due to missing critical clinical information. Patients were enrolled after providing informed consent. The study was approved by the Institutional Ethics Committee of Tongji Hospital of Tongji University. No formal sample-size calculation was performed because the study was based on the available recurrent tumor specimens meeting the study criteria.

### Tissue sample processing, RNA extraction, and transcriptome sequencing

Recurrent tumor tissue samples underwent RNA extraction following pathological evaluation. Samples with tumor cell content >50% based on pathological assessment proceeded to subsequent experiments. RNA was extracted from formalin-fixed paraffin-embedded (FFPE) tissue samples using the QIAGEN RNeasy FFPE Kit. Extracted RNA underwent quality assessment for purity, concentration, and integrity, with qualified RNA used for library construction. Library construction was performed using a poly(A) enrichment-based mRNA library preparation protocol for mRNA enrichment, cDNA synthesis, and amplification. After passing quality control, libraries were subjected to high-throughput sequencing on the Illumina platform.

### Sequencing data quality control, alignment, and expression quantification

Quality control assessment was performed on raw sequencing data, including metrics such as sequencing error rate, data volume, and alignment rate. After passing quality control, reads were filtered and aligned to the human genome reference sequence. Gene-level counting and expression normalization were completed to obtain a gene expression matrix. The processed expression matrix used for downstream analyses was normalized to transcripts per million (TPM) and log2-transformed for statistical analysis and visualization.

### Molecular and immune subtyping

Unsupervised clustering was performed based on the transcriptomic expression matrix. Genes with high expression variability were selected for clustering modeling. The optimal number of clusters was determined using resampling-based consensus assessment to obtain molecular subtype classifications. Samples were annotated with subtype labels using established classification systems, including European Urology (EU) molecular subtypes, for subsequent subtype-related analyses. Immune subtyping was performed using the Tumor Microenvironment Immune Typing (TMIT) system, classifying samples into TMIT types I–IV. Programmed death-ligand 1 (PD-L1) immunohistochemistry results were recorded as negative or positive. Human epidermal growth factor receptor 2 (HER2) immunohistochemistry results were scored as 0, 1+, 2+, or 3 +.

### Differential expression analysis and functional enrichment analysis

Differential expression analysis, Gene Ontology (GO)/Kyoto Encyclopedia of Genes and Genomes (KEGG) enrichment analysis, and Reactome enrichment analysis were performed using R software. Gene-level expression comparisons generated log2 fold changes and nominal P values, with results visualized using volcano plots to identify candidate genes with prominent between-subtype differences. GO enrichment analysis, KEGG pathway enrichment analysis, and Reactome enrichment analysis were performed separately for upregulated and downregulated gene sets or combined, with results visualized accordingly. These analyses were used for candidate screening and functional characterization rather than confirmatory transcriptome-wide inference.

### Clinical variables and outcome definitions

Treatment strategies were determined by clinical decision-making and were not randomly assigned. Patients were categorized according to actual treatment received into immunotherapy monotherapy (Mono) and combination therapy (Combo) groups, with combination therapy including immunotherapy combined with chemotherapy and/or targeted therapy. Because treatment assignment was non-randomized and combination regimens were heterogeneous, treatment-group analyses were interpreted as descriptive rather than as treatment-effect comparisons.

Treatment response was assessed according to Response Evaluation Criteria in Solid Tumors (RECIST) version 1.1 using serial radiological examinations and clinical records. Baseline imaging was obtained before treatment initiation, and follow-up imaging was generally performed at routine clinical intervals (typically every 8–12 weeks), with additional imaging when clinically indicated. Best overall response (Response) was classified as complete response (CR), partial response (PR), stable disease (SD), or progressive disease (PD). Radiological response assessments reflected routine clinical practice and were not subjected to centralized blinded or independent review. Clinical benefit (Benefit) was an originally recorded binary clinical outcome separate from RECIST best response. Benefit = 1 was assigned for CR or PR, or SD maintained for at least 6 months from treatment initiation without documented radiological or clinical progression; Benefit = 0 was assigned for PD as the best overall response or SD lasting less than 6 months. The assessment window began at treatment initiation and extended through month 6. The designation was based on serial radiology reports, treatment records, and routine follow-up documentation assessed by the treating clinical team and recorded in the clinical dataset. For the present revision, the recorded designations were verified against the source records, and no patient was reclassified. Thus, Benefit was not a simple recoding of the four RECIST categories because SD was classified according to its duration.

The survival outcome was progression-free survival (PFS), measured from initiation of the treatment regimen evaluated in this study to the first documented radiological or clinical progression/recurrence. PFS time and event status were determined from follow-up records. Patients without a documented progression/recurrence event were censored at the date of the last disease assessment. Smoking history was recorded as ever smoker (yes/no). Tumor type was classified as non-muscle-invasive bladder cancer (NMIBC) or muscle-invasive bladder cancer (MIBC) based on clinicopathological records.

### Statistical analysis

Statistical analyses were performed with R software. Continuous variables were presented as median and interquartile range (IQR). Comparisons between two groups for continuous variables were performed using the Wilcoxon rank-sum test, and comparisons among multiple groups were performed using the Kruskal-Wallis test. Categorical variables were presented as counts and percentages. Between-group comparisons for categorical variables were performed using Fisher’s exact test to calculate *P* values. For analyses using categorical MAGEA4 expression, the cohort median log2TPM value (2.901) was used to define the High and Low groups (n = 10 each). Receiver operating characteristic (ROC) curves were constructed with clinical benefit (Benefit) as the outcome to assess discriminatory performance, and the area under the curve (AUC) was calculated with 95% confidence intervals (CIs). PFS curves were generated using the Kaplan-Meier method, with differences between groups compared using the log-rank test. An univariable Cox proportional hazards model was used to estimate the hazard ratio (HR) and 95% CI for the High versus Low MAGEA4 groups; given the limited number of events, no multivariable survival model was fitted. All tests were two-sided, and *P* < 0.05 was considered statistically significant. Given the small cohort and multiple secondary comparisons, individual P values were interpreted together with effect estimates and the overall pattern of findings. As sensitivity analyses, multiple-testing correction was performed using the Benjamini-Hochberg false-discovery-rate method, and the overall ROC analysis was internally assessed using 2,000 bootstrap resamples.

## Results

### Patient characteristics and overall treatment response profile

A total of 20 patients with recurrent bladder cancer were enrolled in this study. Patient age, sex, smoking history (ever smoker), tumor type (NMIBC/MIBC), and stage distribution for the overall cohort are presented in **Table 1**. Treatment strategies were categorized according to actual regimens received into immunotherapy monotherapy (Mono) and combination therapy (Combo), with 12 patients (60%) in the Combo group and 8 patients (40%) in the Mono group (**Figure 1**). The distribution of best overall response (Response) was as follows: CR in 2 patients (10%), PR in 3 patients (15%), SD in 7 patients (35%), and PD in 8 patients (40%) (**Figure 1**). The Benefit variable used for ROC analysis was analyzed separately from RECIST best response as defined in the Methods. Overall, 9 patients were classified as Benefit = 1 and 11 as Benefit = 0. Among the 7 patients with SD, 4 maintained SD for at least 6 months and were classified as Benefit = 1, whereas 3 had SD lasting less than 6 months and were classified as Benefit = 0.

**Table 1:** Baseline clinical characteristics of the recurrent bladder cancer cohort (N = 20).

Variable	Overall (N = 20)
Age, years	69.0 [64.5, 79.2]
Sex	
Female	12 (60%)
Male	8 (40%)
Stage	
I	5 (25%)
II	3 (15%)
III	6 (30%)
IV	6 (30%)
Ever smoker	
Yes	12 (60%)
No	8 (40%)
Tumor category (NMIBC/MIBC)	
NMIBC	8 (40%)
MIBC	12 (60%)
Treatment strategy (Mono/Combo)	
Mono	8 (40%)
Combo	12 (60%)

Continuous variables are presented as median [interquartile range], and categorical variables are presented as n (%).

Treatment strategy was categorized as immunotherapy monotherapy (Mono) and combination therapy (Combo).

**Figure 1:**
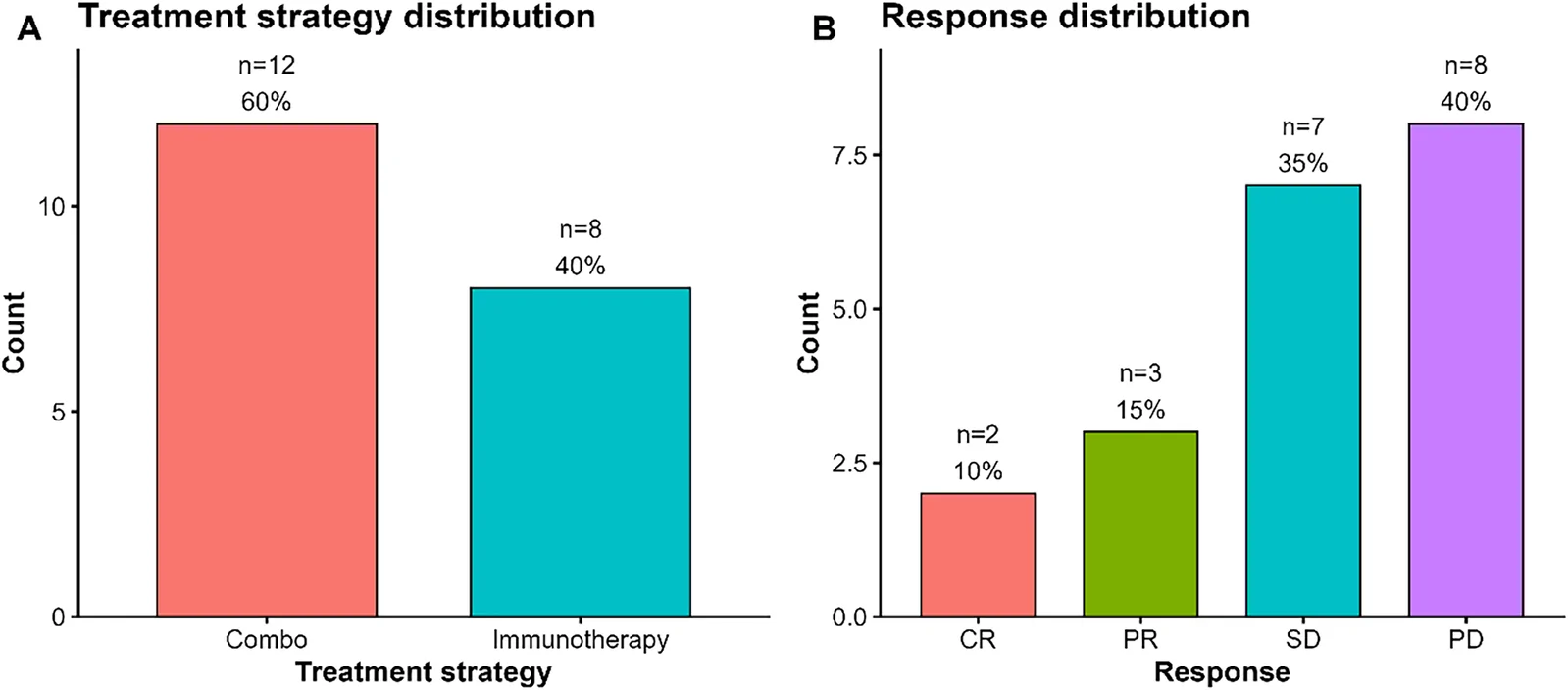
Cohort overview: treatment strategy and best response distribution. **(A)** Distribution of treatment strategies in the full cohort (N = 20). **(B)** Distribution of best response in the full cohort (N = 20), categorized as CR, PR, SD, and PD. Bars indicate counts, and annotations show the corresponding n and percentage.

### Differential expression profile of EU subtypes (luminal papillary vs. others)

To identify molecular differences between EU subtypes, transcriptomic expression profiles were compared between the Luminal papillary group and the Others group, with Others comprising Luminal unstable, Basal/squamous, and Stroma-rich. A volcano plot was generated to visualize the differential expression profile (**Figure 2**), with -log10(*P* value) on the y-axis and log2(fold change) on the x-axis. Using screening thresholds of fold change > 2 and nominal *P* < 0.05, we identified genes with prominent between-subtype expression differences and annotated representative candidates.

**Figure 2:**
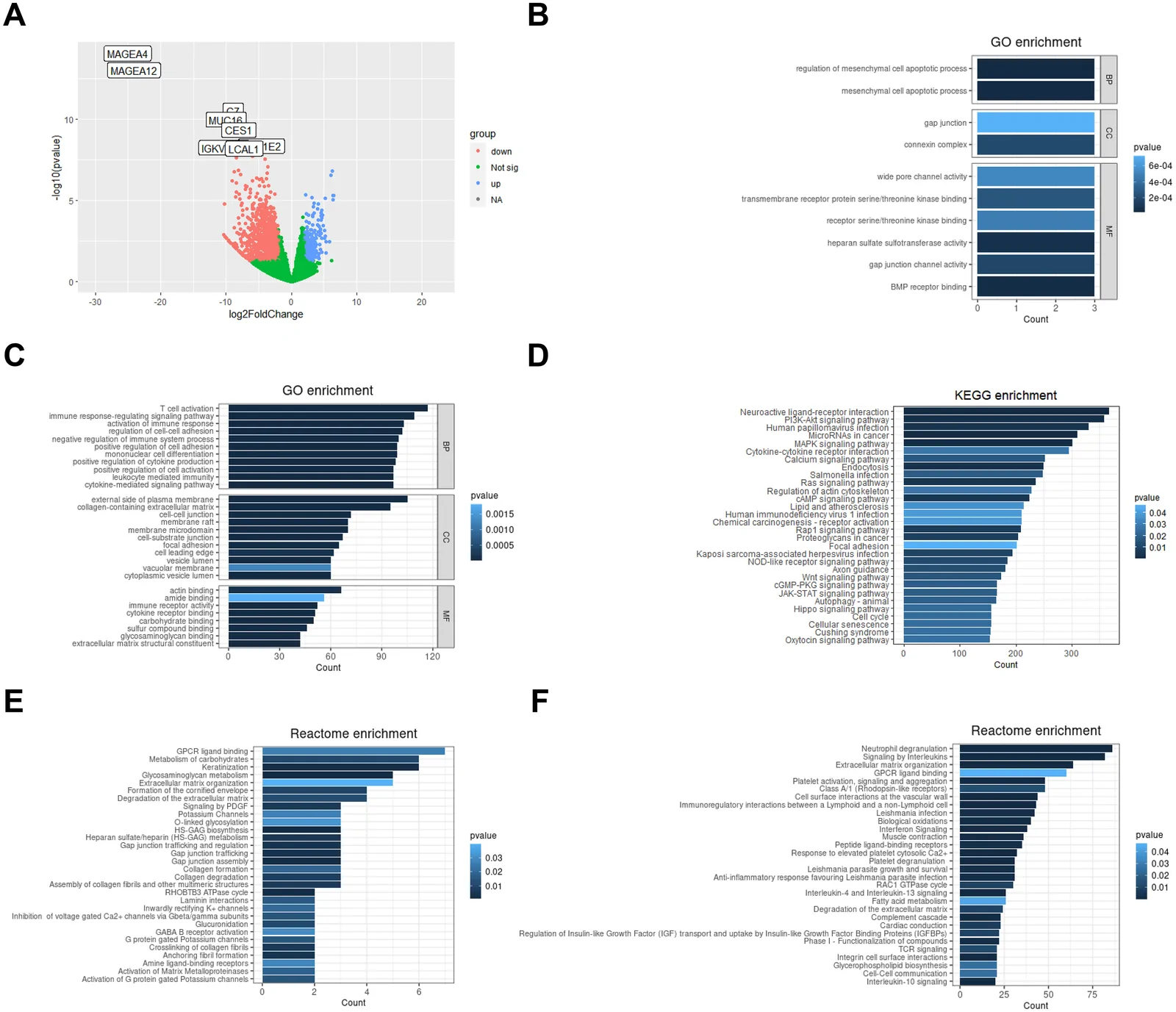
Differential expression and functional enrichment analysis between EU molecular subtypes. Comparison: Luminal papillary vs. the Luminal unstable, Basal/squamous, and Stroma-rich subtypes. **(A)** Volcano plot of differential gene expression. Candidate genes were screened using fold change > 2 and nominal *P* < 0.05. Representative genes with prominent expression differences are labeled. **(B)** GO enrichment analysis of upregulated genes, covering BP, CC, and MF categories. Color represents nominal enrichment *P* value. **(C)** GO enrichment analysis of downregulated genes (BP, CC, MF); color represents nominal enrichment *P* value. **(D)** KEGG pathway enrichment analysis based on the combined DEG set (upregulated and downregulated genes); color represents nominal enrichment *P* value. **(E)** Reactome pathway enrichment analysis of upregulated genes; color represents nominal enrichment *P* value. **(F)** Reactome pathway enrichment analysis of downregulated genes; color represents nominal enrichment *P* value.

The 20 genes with the smallest nominal P values in this comparison are summarized in **Table 2**, including MAGEA4, MAGEA12, MUC16, CES1, C4A, HLA-DPA1, CCDC80, SLIT2, and EFEMP1 (**Table 2**). The top genes included members of the cancer-testis antigen family (e.g., MAGEA4, MAGEA12, MAGEA1) and immune-related molecules (e.g., HLA-DPA1, C4A), as well as structural/signaling molecules associated with tumor biology (e.g., SHH, EFEMP1), suggesting multidimensional molecular differences underlying EU subtypes. Notably, the majority of these candidate genes (including MAGEA4, MAGEA12, MUC16, C7, CES1, and HLA-DPA1) exhibited negative log2 fold change values, indicating relatively higher expression in the combined non-luminal papillary subtype group. In contrast, a few genes such as SHH and LY6D showed relatively higher expression in the luminal papillary subtype (log2FC > 0).

**Table 2:** Top 20 genes identified in the EU molecular subtype expression comparison.

Gene name	log_2_FoldChange	Nominal *P* value
MAGEA4	-25.0560148	8.52E-15
MAGEA12	-24.0940383	9.01E-14
C7	-8.90779183	2.79E-11
MUC16	-9.98708525	1.03E-10
CES1	-8.05210975	4.48E-10
AKR1E2	-4.59360152	4.36E-09
IGKV1-17	-10.3335532	5.49E-09
LCAL1	-7.22340578	6.20E-09
C4A	-5.98345202	1.93E-08
GABRP	-8.389034	2.37E-08
CCDC80	-4.0899488	2.76E-08
SLIT2	-3.62635998	8.60E-08
DMRTA1	-8.06767283	1.36E-07
LY6D	6.2485703	1.52E-07
MAGEA1	-8.77375134	1.73E-07
EFEMP1	-3.76782664	1.95E-07
VNN1	-7.77613093	2.39E-07
SHH	6.01177246	2.87E-07
CFTR	-5.31571565	4.03E-07
HLA-DPA1	-4.56597419	4.55E-07

Differential expression analysis was performed between Luminal papillary tumors and the remaining EU molecular subtypes (luminal unstable, basal/squamous, and stroma-rich).

Candidate genes were screened using fold change > 2 and nominal *P* < 0.05. The 20 genes with the smallest nominal P values are listed.

Negative log2FoldChange values indicate higher expression in the combined group of non-luminal papillary subtypes.

### GO/KEGG and reactome enrichment analysis of differentially expressed genes

Functional and pathway enrichment analyses were further performed on the differentially expressed genes (**Figure 2B**). GO enrichment analysis was performed separately for upregulated and downregulated gene sets (**Figure 2B**). Upregulated genes (relatively higher expression in the luminal papillary group) were primarily enriched in cell junction-related and associated structure/function terms, including connexin complex and gap junction (cellular component [CC]), as well as BMP receptor binding, gap junction channel activity, and heparan sulfate sulfotransferase activity (molecular function [MF]). These findings suggest that the luminal papillary subtype exhibits stronger molecular features related to cell-cell junctions and epithelial structure maintenance. Downregulated genes (relatively higher expression in the luminal unstable + basal/squamous groups) were mainly enriched in terms associated with immune activation and immune response regulation (biological process [BP]), including T cell activation, immune response-regulating signaling pathway, activation of immune response, mononuclear cell differentiation, and positive regulation of cytokine production, as well as external side of plasma membrane and membrane raft (CC), and immune receptor activity and cytokine receptor binding (MF). This indicates a more active immune infiltration background in these subtype groups.

KEGG pathway enrichment analysis was performed based on all differentially expressed genes (**Figure 2**). Enriched pathways included neuroactive ligand-receptor interaction, PI3K-Akt signaling pathway, MAPK signaling pathway, microRNAs in cancer, cytokine-cytokine receptor interaction, JAK-STAT signaling pathway, calcium signaling pathway, and Wnt signaling pathway, among multiple tumor-related and signal transduction pathways. This suggests extensive molecular differences between the two subtype groups at the level of receptor signaling, immune regulation, and tumor microenvironment-related pathways.

Furthermore, Reactome enrichment analysis showed a broadly consistent functional pattern (**Figure 2E**). The upregulated gene set was enriched in terms such as gap junction assembly, gap junction trafficking, keratinization, HS-GAG biosynthesis, glycosaminoglycan metabolism, extracellular matrix organization, and signaling by PDGF, consistent with the molecular characteristics of epithelial differentiation, cell-cell communication, and matrix homeostasis maintenance in the luminal papillary subtype. The downregulated gene set was enriched in immune-related terms including neutrophil degranulation, signaling by interleukins, cell surface interactions at the vascular wall, immunoregulatory interactions between a lymphoid and a non-lymphoid cell, interferon signaling, complement cascade, and interleukin-10 signaling, showing high consistency with the immune infiltration enrichment features revealed by GO analysis.

Collectively, the multi-database enrichment analyses suggested a contrast in which the combined non-luminal papillary subtype group showed more prominent immune-activation and inflammatory-infiltration signals, whereas the luminal papillary subtype showed stronger enrichment signals related to epithelial structure maintenance and cell-cell junctions. These enrichment results provided functional context clues for subsequent association analyses of candidate genes with immune phenotypes and clinical outcomes.

### Molecular/immune subtype composition by treatment strategy

To describe the molecular and immune landscape of patients receiving different treatment strategies, we summarized PD-L1 IHC, HER2 IHC, TMIT subtype, binary EU subtype, and EU subtype by treatment group (**Table 3**). The subtype composition and between-group comparisons for different treatment strategy groups are presented in **Table 3**.

**Table 3:** Distribution of molecular subtype and immune-related characteristics according to treatment strategy.

Variable	Overall (N = 20)	Mono (n = 8)	Combo (n = 12)	*P* value
PD-L1 IHC				0.055
Negative	15 (75%)	8 (100%)	7 (58%)	
Positive	5 (25%)	0 (0%)	5 (42%)	
HER2 IHC				0.405
0	4 (20%)	3 (38%)	1 (8%)	
1+	4 (20%)	1 (13%)	3 (25%)	
2+	10 (50%)	3 (38%)	7 (58%)	
3+	2 (10%)	1 (13%)	1 (8%)	
TMIT subtype				0.298
TMIT I	4 (20%)	2 (25%)	2 (17%)	
TMIT II	6 (30%)	2 (25%)	4 (33%)	
TMIT III	6 (30%)	1 (13%)	5 (42%)	
TMIT IV	4 (20%)	3 (38%)	1 (8%)	
EU subtype (binary)				1.000
Luminal papillary	10 (50%)	4 (50%)	6 (50%)	
Others	10 (50%)	4 (50%)	6 (50%)	
EU subtype				0.614
Luminal papillary	10 (50%)	4 (50%)	6 (50%)	
Luminal unstable	3 (15%)	2 (25%)	1 (8%)	
Basal/squamous	5 (25%)	1 (13%)	4 (33%)	
Stroma-rich	2 (10%)	1 (13%)	1 (8%)	

Categorical variables are presented as n (%). *P* values were calculated using Fisher’s exact test. Mono indicates immunotherapy monotherapy; Combo indicates immunotherapy combined with chemotherapy and/or targeted therapy. EU subtype was additionally summarized as a binary variable for downstream analyses, where Others included luminal unstable, basal/squamous, and stroma-rich.

### Association of MAGEA4 expression levels with immune/molecular subtype composition

Patients were further dichotomized at the cohort median MAGEA4 expression level (2.901 log2TPM) into High and Low groups (n = 10 each) to compare immune and molecular subtype composition (**Figure 3**). Compared with the MAGEA4 Low group, the MAGEA4 High group differed in PD-L1 status distribution (*P* = 0.033, **Figure 3**) and TMIT subtype distribution (*P* = 0.021, **Figure 3**). The distribution of EU subtypes also differed between the two groups (*P* = 0.011, **Figure 3**). These findings suggested statistical associations between MAGEA4 grouping and both immune phenotypes and EU molecular subtypes at the compositional level.

**Figure 3:**
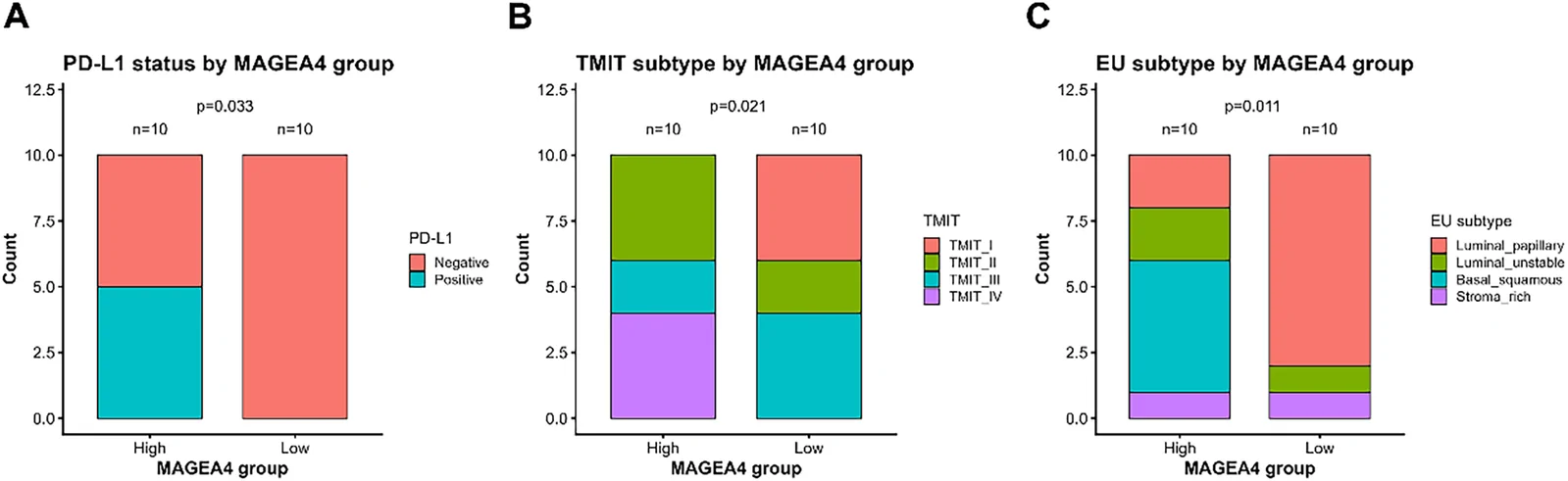
Differences in immune and molecular subtype distributions stratified by MAGEA4 expression. Patients were dichotomized at the cohort median MAGEA4 expression level (2.901 log2TPM) into High and Low groups (n = 10 per group). **(A)** Distribution of PD-L1 IHC status by MAGEA4 group (*P* = 0.033). **(B)** Distribution of TMIT immune subtypes by MAGEA4 group (*P* = 0.021). **(C)** Distribution of EU molecular subtypes by MAGEA4 group (*P* = 0.011). Bars indicate counts, with group sample sizes annotated. *P* values were obtained from Fisher’s exact test as described in the Methods.

### Association of continuous MAGEA4 expression with immune/molecular phenotypes

To further characterize the relationship between MAGEA4 and immune/molecular phenotypes at the continuous expression level, comparative analyses were performed according to PD-L1 status, TMIT subtypes, and the binary EU classification used above (**Figure 4**). Results showed that MAGEA4 expression (log2TPM) differed significantly between PD-L1 negative and positive samples (Wilcoxon test, *P* = 0.0362, **Figure 4**). Significant differences in MAGEA4 expression were also observed among the four TMIT immune phenotypes (Kruskal-Wallis test, *P* = 0.0307, **Figure 4**). Furthermore, after consolidating EU subtypes into Luminal papillary and Others groups following the approach in section 3.2, MAGEA4 expression remained significantly different between the two groups (Wilcoxon test, *P* = 0.0036, **Figure 4**). These results further characterized the association of MAGEA4 expression with immune status and molecular subtype within the same cohort and should not be considered independent validation. After Benjamini-Hochberg correction for multiple testing, the MAGEA4-immune/molecular phenotype associations remained significant, with FDR-adjusted q values ranging from 0.022 to 0.036.

**Figure 4:**
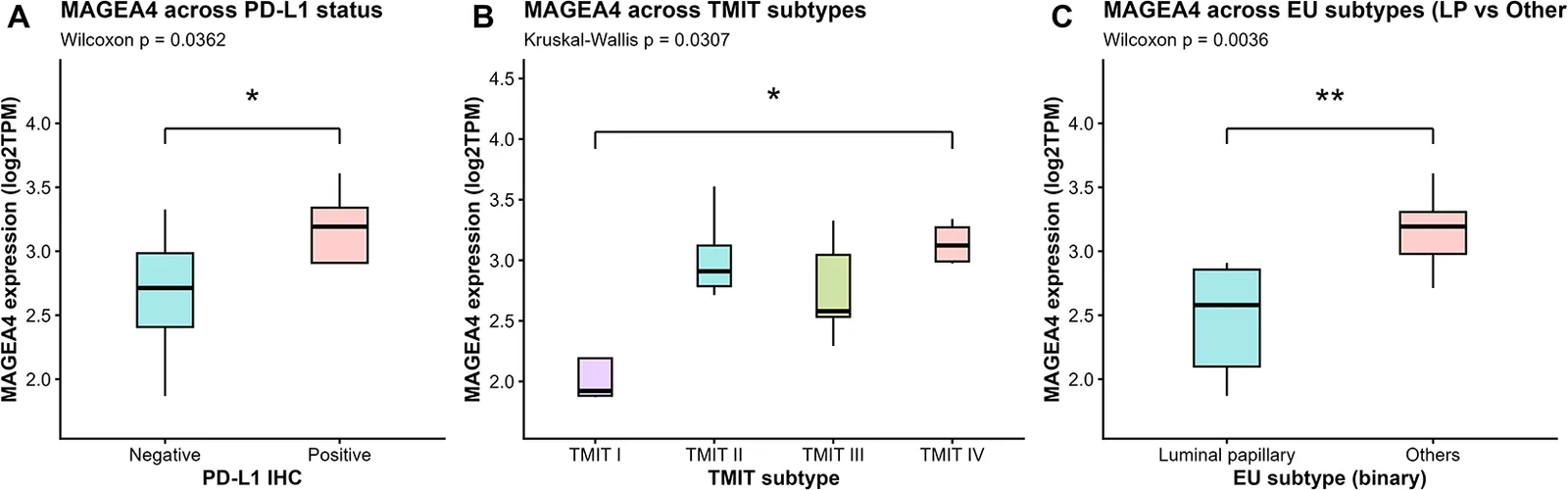
Continuous MAGEA4 expression across immune and molecular phenotypes. **(A)** MAGEA4 expression (log2TPM) according to PD-L1 IHC status. Wilcoxon rank-sum test, *P* = 0.0362. **(B)** MAGEA4 expression (log2TPM) across TMIT immune subtypes. Kruskal–Wallis test, *P* = 0.0307. **(C)** MAGEA4 expression (log2TPM) according to EU molecular subtype (binary grouping: Luminal papillary vs Others). Wilcoxon rank-sum test, *P* = 0.0036. “Others” include the Luminal unstable, Basal/squamous, and Stroma-rich subtypes. Boxplots display the median and interquartile range. Significance annotations are indicated in the figure. These analyses represent within-cohort characterization and not independent validation; multiplicity across the six Figure 3/4 association tests was evaluated using Benjamini-Hochberg FDR correction as described in the Methods. * indicates P < 0.05 and ** indicates P < 0.01.

### Discriminatory performance of MAGEA4 for clinical benefit

ROC curves were constructed based on MAGEA4 expression to evaluate its discriminatory performance for clinical benefit (Benefit) (**Figure 5**). In the overall cohort, the area under the curve (AUC) was 0.798 (95% CI: 0.589–1.000). In the immunotherapy monotherapy subgroup, the AUC was 0.800 (95% CI: 0.454–1.000). In the combination therapy subgroup, the AUC was 0.750 (95% CI: 0.418–1.000) (**Figures 5A–C**). The AUC estimates indicated discrimination of clinical benefit within the present cohort, although the wide confidence intervals, particularly in the subgroups, warrant cautious interpretation. For the overall ROC analysis, 2,000 bootstrap resamples yielded a mean estimated optimism of 0.005, corresponding to an optimism-corrected AUC of 0.793 compared with the apparent AUC of 0.798.

**Figure 5:**
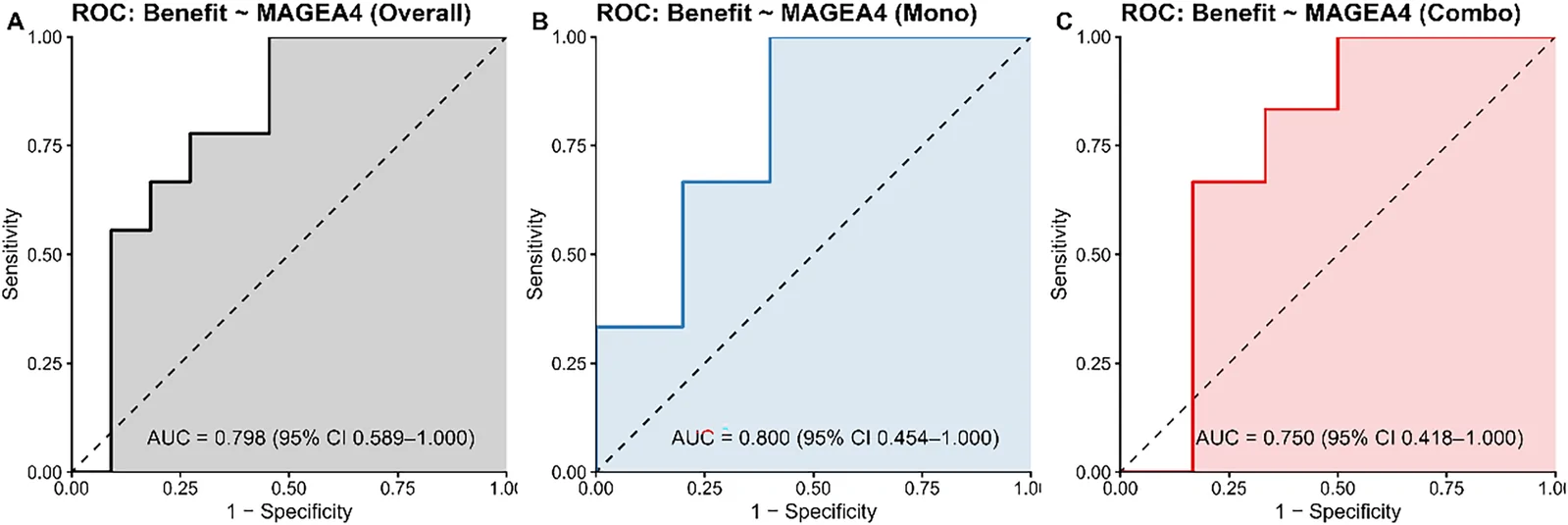
Within-cohort ROC analysis of MAGEA4 for clinical benefit. ROC curves evaluating the discriminatory performance of MAGEA4 expression (log2TPM) for clinical benefit. **(A)** Overall cohort: AUC = 0.798 (95% CI: 0.589–1.000). The bootstrap optimism-corrected overall AUC was 0.793 (2,000 resamples). **(B)** Immunotherapy subgroup: AUC = 0.800 (95% CI: 0.454–1.000). **(C)** Combination therapy (Combo) subgroup: AUC = 0.750 (95% CI: 0.418–1.000). The dashed diagonal line represents the line of no discrimination. Shaded region indicates the area under the curve.

### Survival differences in PFS by MAGEA4 expression

Kaplan–Meier curves demonstrated separation of PFS between the MAGEA4 high-expression and low-expression groups, with a log-rank test *P*-value of 0.036 (**Figure 6**). The unadjusted hazard ratio (HR) for the high-expression group versus the low-expression group was 3.46 (95% CI: 1.02–11.65).

**Figure 6:**
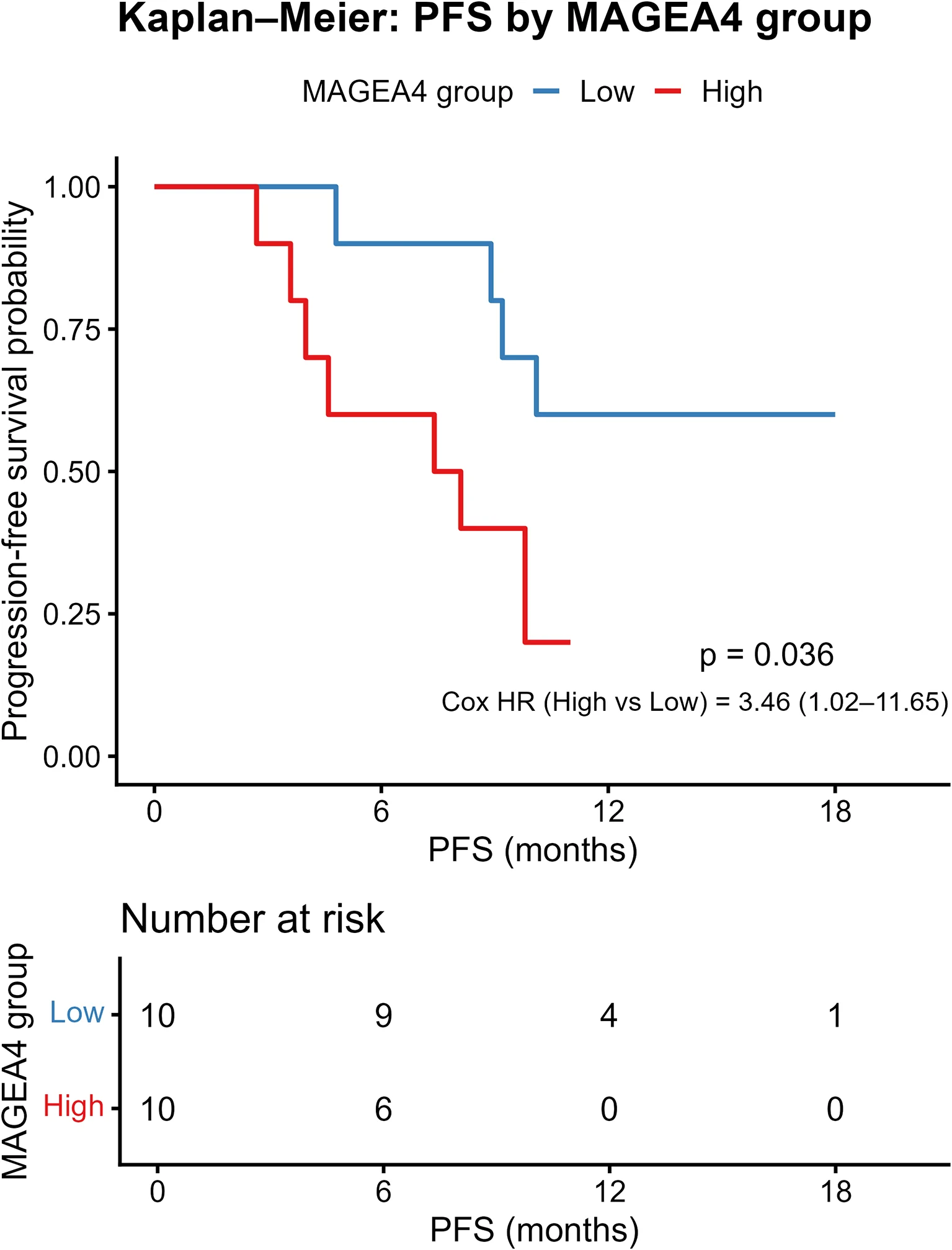
Association between MAGEA4 expression and PFS. Kaplan–Meier curves for PFS stratified by MAGEA4 expression group (High = red, Low = blue), with the number-at-risk table shown below the plot. Log-rank test *P* = 0.036. Univariable Cox proportional hazards model: HR (High vs Low) = 3.46 (95% CI: 1.02–11.65).

The treatment-interaction analysis did not provide evidence of a differential MAGEA4 association by treatment strategy (likelihood-ratio test P = 0.441; **Figure 7**).

**Figure 7:**
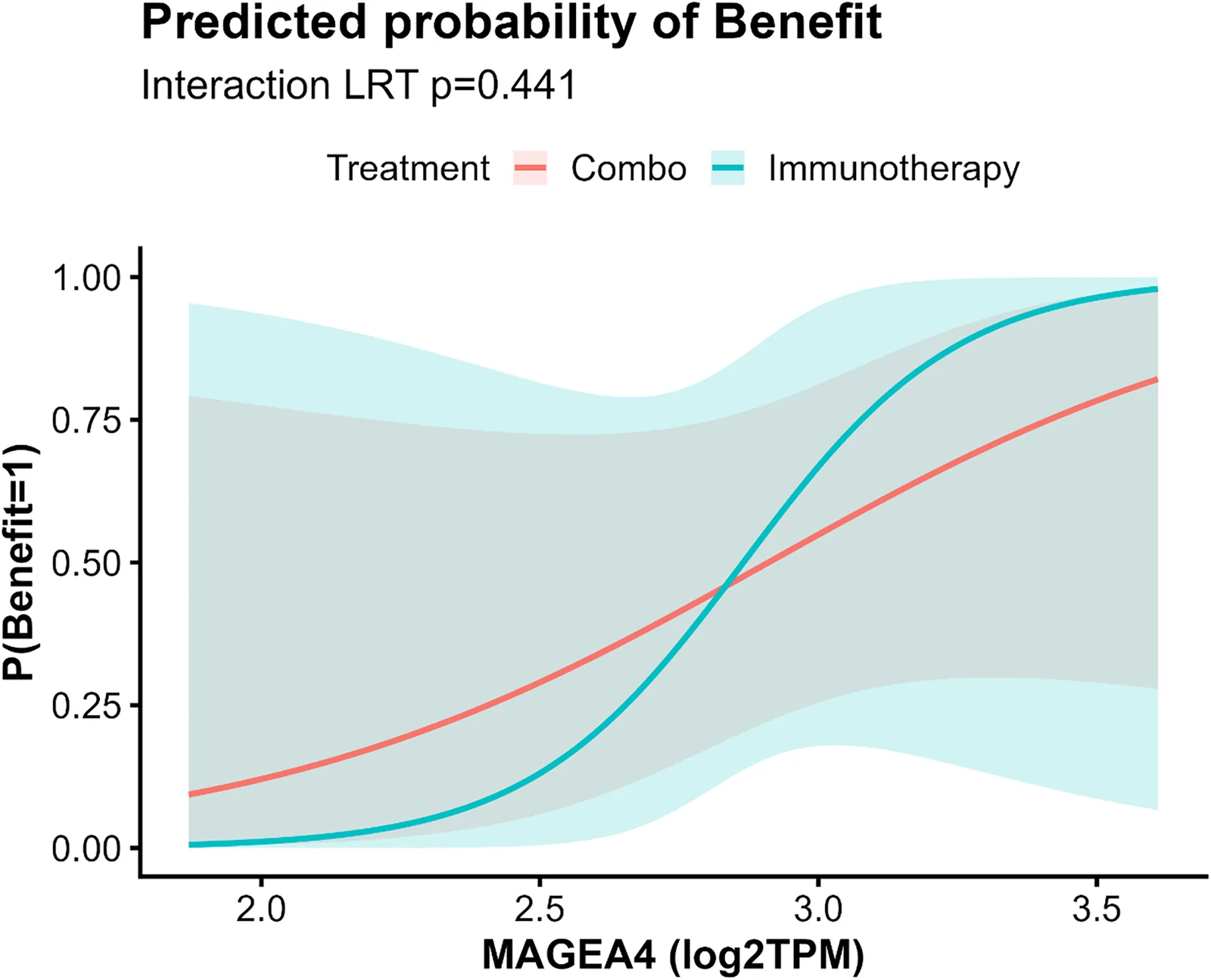
Exploratory interaction analysis between MAGEA4 expression and treatment strategy. Predicted probability curves for clinical benefit according to MAGEA4 expression (log2TPM) under different treatment strategies, derived from a logistic regression model including the MAGEA4 × Treatment interaction term. Shaded areas indicate 95% confidence intervals. The likelihood ratio test (LRT) for the interaction term gave *P* = 0.441.

## Discussion

### Expression characteristics of MAGEA4 in bladder cancer and its subtype context

MAGE-A4 belongs to the cancer-testis antigen (CTA) family. CTA expression in normal somatic cells is strictly restricted to testicular germ cells and placental trophoblast cells, and their aberrant activation, closely associated with promoter region demethylation, has been extensively documented across various tumor types (18, 19). In urothelial tumors, the overall expression rate of CTA family members is relatively high, positioning them as an important source for immunotherapeutic target development (17). A series of studies on bladder cancer have demonstrated that MAGE-A4 protein expression levels increase significantly with rising tumor grade and stage, particularly prominent in high-risk, high-grade transitional cell carcinoma, and are independently associated with cancer-specific survival (15). This expression pattern intensifies with increasing tumor malignancy. Mechanistically, it can be traced to the observation that 5-aza-2’-deoxycytidine-mediated demethylation induces MAGE-A4 reactivation, suggesting epigenetic status as a core regulatory mechanism of CTA expression (20). Furthermore, in upper tract urothelial carcinoma (UTUC), MAGEA family expression is also closely associated with immune microenvironment characteristics and prognosis, further substantiating the systematic significance of CTAs across the entire urothelial tumor spectrum (21). The detection of CTA gene expression in urinary exosomes has been preliminarily explored for non-invasive diagnosis of bladder cancer, reflecting the translational potential of this family (22).

In the present study, comparison of EU molecular subtypes in a recurrent bladder cancer cohort revealed relatively higher MAGEA4 expression in subtypes such as luminal unstable and basal/squamous. This finding aligns directionally with the aforementioned grade/stage-dependent expression pattern: subtypes like luminal unstable and basal/squamous generally exhibit higher genomic instability and epigenetic plasticity at the molecular level, providing a favorable context for demethylation-induced MAGEA4 activation (23, 24).

### Molecular background differences revealed by EU molecular subtyping

This study employed the EU classification system for molecular annotation of the recurrent cohort. GO, KEGG, and Reactome enrichment analyses revealed a highly consistent contrasting pattern between the luminal papillary subtype and the luminal unstable, basal/squamous, and stroma-rich subtypes. Upregulated genes in the former were enriched in pathways related to cell-cell junctions and epithelial structure maintenance (e.g., connexin complex, gap junction, keratinization, extracellular matrix [ECM] organization), whereas the latter were predominantly characterized by enrichment in pathways associated with immune activation and inflammatory infiltration (e.g., T cell activation, neutrophil degranulation, interferon signaling, complement cascade, interleukin signaling). This pattern aligns with the core opposition axis described by multiple independent classification systems: luminal subtypes maintain epithelial differentiation features, while basal/squamous and related subtypes are primarily characterized by stromalization and immune infiltration (9, 25, 26).

Notably, despite variations in the number and nomenclature of subtypes across systems such as TCGA, UNC, MDA, Lund, and EAU, cross-system alignment analyses have demonstrated that the luminal versus basal/squamous opposition remains highly consistent across different classification frameworks, representing the most robust structural feature of bladder cancer molecular heterogeneity (27–30). Regarding treatment response, these molecular background differences carry direct clinical implications: the basal/squamous subtype shows relatively better response to neoadjuvant chemotherapy, whereas luminal subtypes are generally insensitive to platinum-based chemotherapy; luminal subtypes harbor a higher frequency of FGFR3 activating mutations, making targeted therapy more relevant in this subtype; and subtypes characterized by immune infiltration typically exhibit more active responses to ICI therapy (31–34). These findings collectively suggest that molecular subtyping serves not only as a prognostic stratification tool but also as a potential basis for treatment selection, warranting systematic evaluation of its applicability in recurrent cohorts (34, 35).

### Association between MAGEA4 and PD-L1 status: CTA co-expression in an immunologically active background

This study observed a significantly higher proportion of PD-L1 positivity in the MAGEA4 high-expression group compared to the low-expression group (*P* = 0.033). This association can be interpreted from the following perspectives.

At the tumor microenvironment level, upregulation of PD-L1 is typically driven by interferon-gamma (IFN-γ) produced by infiltrating lymphocytes, representing an adaptive response of tumors to immune attack rather than an intrinsic expression characteristic. Demethylation-induced activation of CTAs is associated with genomic instability, which itself can increase tumor immunogenicity—a greater burden of somatic mutations and neoantigens often accompanies more active T cell infiltration, subsequently inducing PD-L1 upregulation. Therefore, the co-expression of MAGEA4 and PD-L1 does not necessarily imply a direct molecular regulatory relationship between them, but more likely reflects that they jointly point to a specific intrinsic tumor phenotype: a subset characterized by immune activity but with concurrent activation of multiple immune escape mechanisms.

At the molecular mechanism level, frequent activating mutations of FGFR3 in luminal bladder cancer degrade PD-L1 protein through the NEDD4-mediated ubiquitination pathway, resulting in generally low PD-L1 expression in the luminal papillary subtype harboring FGFR3 mutations (35). Correspondingly, basal/squamous and luminal unstable subtypes, which have lower frequencies of FGFR3 mutations, exhibit higher PD-L1 expression. The enrichment of high MAGEA4 expression precisely in these non-luminal papillary subtypes aligns with the direction of the present findings.

Furthermore, the PD-1/PD-L1 axis is not the sole immune checkpoint pathway in bladder cancer. The NKG2A/HLA-E axis has been demonstrated to constitute an independent alternative immunosuppressive pathway in bladder cancer, particularly active in PD-L1-positive tumors, suggesting potential systemic limitations of therapies targeting a single checkpoint (36). Intratumoral CD4^+^ T cells can exert direct cytotoxic effects in bladder cancer, and their presence represents a potential factor associated with response to anti-PD-1 immunotherapy (37). These findings collectively indicate that the immunological characteristics of the MAGEA4+/PD-L1+ co-expressing subgroup are complex and should not be simplistically categorized within a binary “immune-hot” or “immune-cold” framework.

### Association between MAGEA4 and TMIT immune subtypes and the multidimensional context of immune checkpoint resistance

This study further observed significant differences in continuous MAGEA4 expression levels among the four TMIT immune subtypes (Kruskal-Wallis *P* = 0.0307). TMIT classification, based on the combination of tumor cell PD-L1 expression and CD8^+^ T cell density, represents a commonly used clinical parameter framework for assessing the bladder cancer immune microenvironment. The differential distribution of MAGEA4 expression across TMIT subtypes further supports its cross-level association with immune phenotypes.

Regarding resistance mechanisms to immune checkpoint therapy, existing evidence presents a complex picture involving multiple parallel pathways. Activation of endogenous interleukin-6 (IL-6) signaling in CD8^+^ T cells can promote resistance to anti-PD-L1 therapy (38). Elevated systemic and tumor-associated interleukin-8 (IL-8) levels are associated with reduced clinical benefit from ICI (39). S100A5 can attenuate the efficacy of anti-PD-L1/PD-1 therapy by inhibiting relevant pathways (40). Acquired resistance models have also revealed the coexistence of multiple escape mechanisms (41). This body of evidence suggests that treatment response may not be fully captured by a single molecular feature and supports multidimensional assessment of the tumor immune context. In the present cohort, the associations of MAGEA4 with TMIT subtype and PD-L1 status suggest that it may provide complementary molecular information alongside established immune phenotypes. Whether this information improves treatment stratification requires evaluation in independent, adequately sized cohorts.

### Prognostic association of MAGEA4 and the translational context of MAGE-A4-directed TCR-T therapy

PFS analysis showed shorter PFS in the MAGEA4 high-expression group than in the low-expression group (log-rank *P* = 0.036; unadjusted HR = 3.46, 95% CI: 1.02–11.65). This finding suggests that high MAGEA4 expression may be associated with more aggressive tumor biology: high MAGEA4 expression is enriched in more aggressive subtypes such as luminal unstable and basal/squamous, which are inherently associated with worse natural history in urothelial tumors (24, 42). High MAGEA4 expression itself represents one phenotypic marker of high-stage, high-grade tumors (15, 16). Therefore, the observed PFS association may partly reflect underlying tumor aggressiveness and other clinicopathological or treatment-related factors. These findings support the potential prognostic relevance of MAGEA4 but do not establish it as an independent prognostic factor.

From a therapeutic target perspective, MAGE-A4 already possesses a substantial immunological evidence base. Early studies identified multiple MAGE-A4/4a restricted peptides recognized by cytotoxic T lymphocytes, spanning various MHC alleles including HLA-A1, HLA-A24, and HLA-B37 (43–45). HLA-DR1-restricted MAGE-A3 peptides recognized by CD4^+^ T cells have also been described, suggesting that CTAs can simultaneously activate both CD4 and CD8 arms of the immune response (46). Building on this foundation, the phase 1 ADP-A2M10 study evaluated MAGE-A10-targeted SPEAR T cells in MAGE-A10-positive solid tumors, including urothelial tumors (47). MAGE-A4-directed TCR-T therapy has since achieved clinical translation in synovial sarcoma; afamitresgene autoleucel (TECELRA) received FDA accelerated approval in 2024 for selected patients with unresectable or metastatic synovial sarcoma who meet HLA and MAGE-A4 expression requirements (19). Furthermore, other novel immunotherapies for genitourinary malignancies, such as NK-CAR therapy, are also exploring targeting strategies against germ cell-related antigens, indicating a broad research landscape in this field (48).

These developments provide a rationale for investigating MAGEA4 in molecularly defined urothelial cancer subsets, but tumor MAGEA4 RNA expression alone is insufficient to establish eligibility for MAGE-A4-directed TCR-T therapy, which also depends on antigen and HLA requirements. Accordingly, the present findings should be viewed as a basis for future translational studies rather than as a patient-selection strategy. Against the backdrop of rapid advances in immunotherapy for genitourinary tumors, evaluating how MAGEA4-defined phenotypes relate to emerging immunotherapeutic strategies remains a relevant translational question (49).

### Limitations and future perspectives

This study has several limitations. The relatively small sample size (n = 20) and non-randomized treatment assignment determined by clinical decisions may introduce confounding bias. The wide confidence intervals, particularly in stratified subgroup analyses, warrant cautious interpretation of statistical results, and external validation is necessary to assess the robustness of current findings. The presence of multiple subtype categories with limited case numbers reduces the precision of contingency-table comparisons. Gene and pathway analyses were conducted using the processed TPM expression matrix with nominal screening thresholds; a multiplicity-adjusted count-based reanalysis was not performed, which increases the risk of false-positive findings. In addition, MAGEA4 was identified and subsequently evaluated within the same cohort, so the subtype analyses do not constitute independent validation. Response, Benefit, and follow-up events were derived from routine clinical and radiological records without centralized blinded review, which may introduce measurement variability. The ROC estimates were obtained in the same cohort and the PFS HR was unadjusted; therefore, these results require independent confirmation. The biological mechanisms underlying the relationships between MAGEA4, immune phenotypes, and treatment outcomes also require experimental validation.

Future studies should include larger independent cohorts, reproducible count-based transcriptomic workflows with appropriate multiple-testing control, and sufficiently powered multivariable analyses to determine whether MAGEA4 provides information beyond established clinicopathological and molecular features. Functional studies integrating quantitative immune profiling may further clarify the biological basis of the observed associations.

## Conclusion

In this single-center recurrent bladder cancer cohort, EU molecular subtype comparison identified subtype-related expression patterns and associated immune- and tumor-related pathway characteristics. MAGEA4 expression was associated with PD-L1 status, TMIT immune subtypes, and EU molecular subtypes and showed within-cohort discriminatory performance for clinical benefit. Further survival analysis indicated that high MAGEA4 expression was associated with poorer PFS. These findings support MAGEA4 as a candidate molecular stratification marker linking molecular subtype, immune phenotype, and clinical outcome. Its clinical utility and treatment-specific relevance require independent validation in larger cohorts.

## Data Availability

The datasets generated and analyzed in this study are not publicly available because participant consent and institutional ethics approval did not permit unrestricted public deposition of individual-level genomic data. De-identified processed expression and clinical data may be made available to qualified researchers from the corresponding author upon reasonable request, subject to applicable ethical and institutional approval and an appropriate data-use agreement.
